# Distinct RPA functions promote eukaryotic DNA replication initiation and elongation

**DOI:** 10.1093/nar/gkad765

**Published:** 2023-09-22

**Authors:** Alexandra M Pike, Caitlin M Friend, Stephen P Bell

**Affiliations:** Howard Hughes Medical Institute, Massachusetts Institute of Technology, Department of Biology, Cambridge, MA 02139, USA; Massachusetts Institute of Technology, Department of Biology, Cambridge, MA 02139, USA; Howard Hughes Medical Institute, Massachusetts Institute of Technology, Department of Biology, Cambridge, MA 02139, USA

## Abstract

Replication protein A (RPA) binds single-stranded DNA (ssDNA) and serves critical functions in eukaryotic DNA replication, the DNA damage response, and DNA repair. During DNA replication, RPA is required for extended origin DNA unwinding and DNA synthesis. To determine the requirements for RPA during these processes, we tested ssDNA-binding proteins (SSBs) from different domains of life in reconstituted *Saccharomyces cerevisiae* origin unwinding and DNA replication reactions. Interestingly, *Escherichia coli* SSB, but not T4 bacteriophage Gp32, fully substitutes for RPA in promoting origin DNA unwinding. Using RPA mutants, we demonstrated that specific ssDNA-binding properties of RPA are required for origin unwinding but that its protein-interaction domains are dispensable. In contrast, we found that each of these auxiliary RPA domains have distinct functions at the eukaryotic replication fork. The Rfa1 OB-F domain negatively regulates lagging-strand synthesis, while the Rfa2 winged-helix domain stimulates nascent strand initiation. Together, our findings reveal a requirement for specific modes of ssDNA binding in the transition to extensive origin DNA unwinding and identify RPA domains that differentially impact replication fork function.

## Graphical Abstract

**Graphical Abstract:**
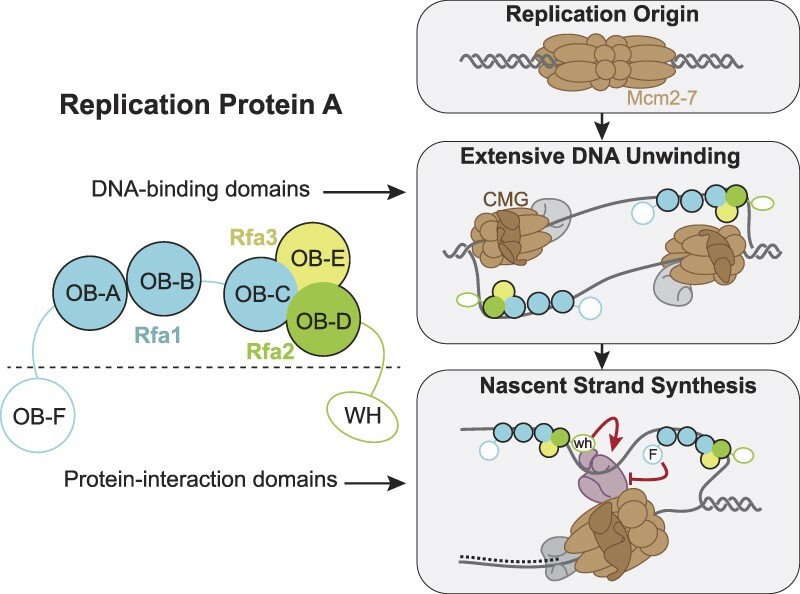

## INTRODUCTION

Eukaryotic DNA replication requires the progressive assembly of protein complexes at origins of replication (1). Origins are licensed during the G1 phase of the cell cycle, when two hexameric Mcm2-7 helicases are loaded onto each origin as a head-to-head double hexamer encircling double-stranded DNA (dsDNA) (2–5). Upon S-phase entry, S-phase cyclin-dependent kinase (S-CDK) and Dbf4-dependent kinase (DDK) along with additional replication proteins activate the helicase for DNA unwinding (6). Once activated, the helicases act separately to bidirectionally unwind the origin and adjacent DNA (7). The activated helicases and the single-stranded DNA (ssDNA) they generate recruit the remainder of the DNA synthesis machinery to form a bidirectional pair of replication forks (8).

Helicase activation is the committed step of replication initiation and involves recruitment of key proteins to the helicase and origin DNA unwinding. Nine proteins, referred to as activation factors, are sufficient to activate loaded helicases *in vitro* (9). Eight of the activation factors coordinate the recruitment of Cdc45 and GINS to Mcm2-7 to form the CMG (Cdc45/Mcm2-7/GINS) complex (1). Cdc45 and GINS directly activate both the ATPase and helicase activity of Mcm2-7 (10), converting the Mcm2-7 complex to the active replicative DNA helicase, CMG. Finally, Mcm10 further primes the helicase for DNA unwinding (11–14). Experiments omitting individual activation factors reveal several steps in origin DNA unwinding (12,15). First, CMG formation results in a small amount of DNA melting (∼6–7 bp per helicase). Addition of Mcm10 stimulates CMG to unwind a limited amount of additional DNA (10–15 bp). Finally, the eukaryotic single-stranded DNA binding protein (SSB) replication protein A (RPA) is required for extensive DNA unwinding (12).

**Figure 1.**
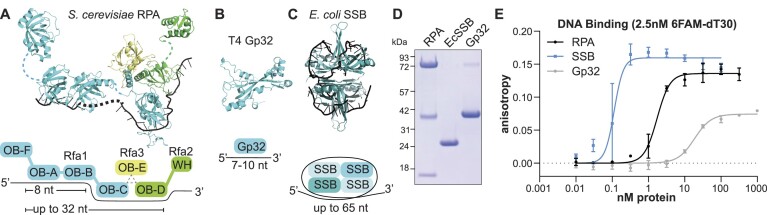
Replicative single-stranded DNA binding proteins vary in their structure and affinity for ssDNA. (A–C) Structure and cartoon diagram of the subunits and DNA-binding modes of (**A**) the RPA heterotrimer, constructed with structures of OB-F (5OMB), OB-A and OB-B bound to ssDNA (1JMC), the OB-C/D/E bound to ssDNA (6I52) and Rfa2-WH (1Z1D), connected by dashed lines representing unresolved protein and DNA sequence (Rfa1, cyan; Rfa2, green; Rfa3, yellow; ssDNA, black). OB = oligonucleotide/oligosaccharide-binding fold; WH = winged-helix domain, (**B**) T4 Gp32 monomer (1GPC) and (**C**). *E. coli* SSB (EcSSB) homotetramer bound to ssDNA (1EYG). Long lozenge shapes in cartoons represent OB-fold domains. (**D**) Coomassie stained SDS-PAGE gel of purified RPA, EcSSB and Gp32. (**E**) Fluorescence anisotropy measured with 2.5 nM 6-FAM-labeled oligo-dT30 and the indicated concentrations of RPA, EcSSB or Gp32. Anisotropy values were plotted against the protein concentrations and fit to the Hill equation. Apparent *K*_d_ values: Gp32 ∼18 nM, RPA ∼ 1.7 nM, EcSSB ∼ 0.11 nM (see also Supplemental Table S1).

RPA is an essential SSB in eukaryotes that binds ssDNA in a sequence-nonspecific manner (16–18). In addition to its requirement for extensive origin DNA unwinding (12) and stimulation of CMG DNA unwinding activity (19), RPA also protects ssDNA and coordinates DNA damage signaling and DNA repair (20). RPA is a heterotrimeric protein that has a conserved domain structure across eukaryotes (Figure 1A) (17). The largest subunit, called Rfa1 in *Saccharomyces cerevisiae*, consists of four oligonucleotide/oligosaccharide-binding (OB)-fold domains, three of which bind DNA (OB-A, OB-B and OB-C) and another that functions as a protein-interaction domain (OB-F) (20,21). The middle subunit, Rfa2, contributes a fourth DNA-binding OB-fold domain (OB-D) and a C-terminal winged-helix (WH) domain. Rfa3 is the smallest subunit, consisting of a single OB-fold (OB-E) that is required for trimerization of the complex but does not bind DNA. The three proteins form a tight, structurally-conserved trimerization core between OB-folds C, D and E (Figure 1A) (22).

RPA associates with ssDNA in a highly dynamic manner. These dynamics have been studied both with isolated domains and in the context of the full-length protein (23). RPA can adopt multiple ssDNA-binding conformations with varying affinities using its four DNA-binding domains (OB-A through -D; Figure 1A). Depending on the domains involved, RPA can bind as few as 8 nucleotides and as many as 32 nucleotides of ssDNA (24,25). Binding affinities of RPA and its sub-domains have been reported in the nanomolar to sub-nanomolar range (20). Although cooperative RPA-ssDNA binding was initially observed (26), more recent evidence suggests RPA cooperativity only occurs when RPA is phosphorylated during the DNA-damage response (27). Structural studies show that the different RPA DNA-binding domains bind ssDNA in different conformations. OB-A and OB-B bind linear stretches of DNA (28), whereas, the structure of the trimerization core shows a dramatic local bend in the ssDNA as it wraps around the OB-C and OB-D domains (27,29). Structural studies have yet to determine the relative positions of the different elements of RPA presumably due to their intrinsically dynamic behavior.

SSBs are present in all domains of life as well as a subset of viruses, but these proteins display a wide variety of structures and ssDNA-binding affinities (reviewed in (30)). The first SSB characterized was Gp32 (Gene 32 protein), which is a monomeric 34 kDa protein that acts during T4 bacteriophage replication (Figure 1B). Gp32 consists of a single OB-fold that can bind up to 10 nucleotides of ssDNA with a *K*_d_ in the 10 nanomolar range (31,32). The canonical bacterial SSB was initially identified in *Escherichia coli* (referred to here as EcSSB), and binds DNA as a homotetramer (Figure 1C) (33). A single EcSSB tetramer has four OB-fold DNA-binding domains that transition between multiple DNA-binding conformations, wrapping as few as 35 and up to 65 nucleotides around its core (34). EcSSB DNA-binding affinity is higher than Gp32 and RPA, with dissociation constants in the sub-nanomolar range (35,36). Unlike RPA, both Gp32 and EcSSB exhibit strong cooperative DNA binding (37,38). Despite their different ssDNA-binding properties, RPA, Gp32 and EcSSB each play an essential role during DNA replication in their cognate organism.

Much of our understanding of RPA function during eukaryotic DNA replication comes from studies of simian virus 40 (SV40) DNA replication in human cells. This viral replication fork uses the SV40 large T-antigen (LTag) as the replicative helicase and a subset of the human DNA synthesis machinery to replicate the SV40 genome. Studies of SV40 DNA replication *in vitro* have shown that neither DNA unwinding nor DNA synthesis occur in the absence of RPA (39–41). Additionally, human RPA has a unique function during SV40 DNA replication, but not DNA unwinding, that cannot be performed by other SSBs, including yeast RPA (42,43). Specific interactions between human RPA and the SV40 replication machinery are presumed to mediate the specificity of this function. For example, human but not yeast RPA can bind LTag to promote primosome assembly (44). Further, RPA can stimulate Pol-α/primase activity (43,45,46) and promote the Pol-α/primase to Pol δ polymerase switch during lagging-strand synthesis (47–49). However, RPA interactions with replication proteins have not been addressed at a fully eukaryotic DNA replication fork.

Here, we investigate the roles of RPA during eukaryotic DNA replication initiation and elongation. Because RPA has not been observed to interact with CMG helicase, we asked if DNA unwinding requires RPA specifically or only its ssDNA-binding activity. By testing either Gp32, EcSSB or RPA mutants in reconstituted origin unwinding assays, we found that neither multiple ssDNA-binding domains nor high ssDNA-binding affinity is sufficient to facilitate extensive origin DNA unwinding. Instead, a specific arrangement of DNA-binding but not protein-protein interaction domains is required for this event. In contrast, using reconstituted replication assays, we found that, unlike origin unwinding, the replication fork requires two RPA domains in addition to its ssDNA-binding domains for normal DNA synthesis. Analysis of mutants individually lacking each of these domains provides insights into the steps in replication that they participate in.

## MATERIALS AND METHODS

### RPA expression plasmids and strains

RPA was expressed and purified from yAE31(9). RPA protein-interaction domain mutants were made by mutating the wild-type expression plasmids to generate pRS303-Gal1,10-CBP-Rfa1^ΔOB-F^/Gal4 and pRS306-Gal1,10–3xFlag-Rfa2^ΔWH^/Rfa3. CBP-Rfa1^ΔOB-F^ contains coding sequence for Rfa1 residues 131–621, and 3xFlag-Rfa2^ΔWH^ contains coding sequence for Rfa2 residues 1–202. The respective plasmids were integrated into yRH101 to generate yAP05 (CBP-Rfa1^ΔOB-F^, Rfa2, Rfa3); yAP17 (CBP-Rfa1, 3x-Flag-Rfa2^ΔWH^, Rfa3); yAP18 (CBP-Rfa1^ΔOB-F^, 3x-Flag-Rfa2^ΔWH^, Rfa3). Other RPA mutants were expressed in bacteria. To make RPA^AB^, the coding sequence for CBP-OB-A-OB-B was ordered as a gBlock (IDT) and cloned into the pET3aTr backbone for expression in *E. coli*. RPA^ABAB^ was generated by PCR amplifying OB-A and OB-B and the linker that joins to the N-terminal boundary of OB-C (OB-AB + linker) followed by Gibson assembly into the RPA^AB^ plasmid. To express the trimerization core, the p11d-tscRPA-30MxeHis6 plasmid (50) was modified by truncating the coding sequences for Rfa1 and Rfa2 to express only OB-C (Rfa1_442-621_) and OB-D (Rfa2_232-182_) respectively. The resulting plasmid contains an inducible Rfa1-OB-C, Rfa2-OB-D and Rfa3 coding sequences with an intein, chitin-binding domain and a 6xHis tag at the Rfa2 C-terminus. All mutations were confirmed by Sanger sequencing. Additional details on expression constructs and yeast strains can be found in Supplementary Tables S2 and S3.

### Protein expression and purification

Purified *E. coli* SSB (Sigma-Aldrich S3917) and T4 Gene 32 Protein (Gp32; Roche 10972983001) were obtained from commercial vendors. *Saccharomyces cerevisiae* RPA, RPA^ΔOB-F^, RPA^ΔWH^ and RPA^ΔOB-FΔWH^ were purified from yeast and RPA^AB^, RPA^ABAB^ and RPA^Tri-C^ were purified from *E. coli* as described below. RPA purified from both yeast and bacteria have been shown to support *in vitro* DNA replication (9,14)

Wild-type RPA (CBP-Rfa1, Rfa2 and Rfa3) was expressed and purified from yAE31 using calmodulin and HiTrap Heparin columns as described in (9). RPA^ΔOB-F^ was expressed and purified from yAP05 using the same protocol. Briefly, 8 L of logarithmic culture were alpha-factor arrested and RPA expression was induced with galactose for 3.5 hours. Cells were harvested and ground into powder which was then thawed into Buffer C (25 mM Tris–HCl pH 7.2, 10% glycerol, 1 mM DTT) with 500 mM NaCl. Lysate was clarified by ultracentrifugation (200,000 x g for 90 min), then supplemented with 2 mM CaCl_2_ and bound to a 1 ml calmodulin-affinity column. RPA was eluted with Buffer C supplemented with 200 mM NaCl, 2 mM EDTA and 2 mM EGTA. RPA-containing fractions were pooled, dialyzed against Buffer C with 50 mM NaCl and 1 mM EDTA, and applied to a 1 ml HiTrap Heparin column (Cytiva) equilibrated in the same buffer. RPA was eluted with a 30 column volume (CV) gradient from 50 mm to 1 M NaCl in Buffer C + 1mM EDTA. RPA-containing fractions were collected, snap-frozen on liquid nitrogen and stored at −80°C until use.

For purification of RPA^ΔWH^, lysate from 8L of yAP17 (CBP-Rfa1, 3xFlag-Rfa2-ΔWH and Rfa3) was prepared as described for RPA and then bound to 1ml Flag resin (Sigma) equilibrated in Buffer C with 100 mM NaCl, washed and eluted with Buffer C supplemented with 100 mM NaCl and 0.3 mg/ml 3xFlag peptide. RPA-containing fractions were pooled, concentrated and applied to S200 Increase 10/300 column (Cytiva) equilibrated in Buffer C with 200 mM NaCl and 1 mM EDTA. Stoichiometric RPA complexes were snap frozen and stored at −80°C. RPA^ΔOB-FΔWH^ was expressed and purified from 16 L of yAP18 using the approach for WT RPA, except a flag affinity step (as described for RPA^ΔWH^) was added between the calmodulin and heparin columns to enrich for complexes that contained both mutant subunits.

For RPA^AB^ and RPA^ABAB^, the appropriate expression plasmid was transformed into Rosetta(DE3)pLysS cells, expanded to 2 l and induced with 1 mM IPTG during mid-log phase and incubated at 16°C overnight. Cells were pelleted and lysed by sonication in C/500 (25 mM Tris–HCl pH 7.2, 10% glycerol, 1 mM DTT, 500 mM NaCl and 1 mM EDTA). Lysates were then purified by calmodulin-affinity and heparin columns as described for WT RPA.

For RPA^Tri-C^, p11d-scTriC was transformed into Rosetta(DE3)pLysS and expressed and purified as in (14), except after the Ni-NTA and chitin columns, the eluate, which had a significant excess of Rfa2, was pooled and passed over a heparin column. The monomeric Rfa2 saturated the heparin column and Tri-C was concentrated in the flow-through. The flow-through was collected and Tri-C was further purified by binding to a 1 ml MonoQ column (Cytiva) equilibrated in Buffer C with 50 mM NaCl and 1 mM EDTA, then eluted with a 30 CV gradient from 50 mM to 1 M NaCl. Fractions containing trimeric RPA were further purified further by size exclusion on S75 Increase column (Cytiva) in Buffer C with 200 mM NaCl and 1 mM EDTA, snap-frozen and stored at −80°C.

For reconstituted plasmid unwinding and replication assays, Mcm2-7/Cdt1 and ORC complexes were purified as described previously (51). Cdc6 was purified as described in (52). DDK, S-CDK, Sld3/7, Sld2, Dpb11, GINS, Mcm10 and Pol ϵ were purified as described in (14) and Cdc45 was purified according to (53).

### Fluorescence polarization assays

For RPA/EcSSB/Gp32 DNA-binding, serial 2-log dilutions of the respective SSB were prepared in 30 mM HEPES pH 7.5, 30 mM NaCl, 0.25 mM EDTA, 10% glycerol, 0.01% NP-40, 1 mM DTT. Proteins were then mixed with 6-FAM-labeled oligo-dT30 (IDT) to a final concentration of 2.5 nM supplemented with 0.5 M NaCl (54). The protein–DNA mix was incubated for at least 30 min at room temperature to reach equilibrium (55). Three technical replicates were plated in a black 384-well nonbinding microplate (Greiner Bio-One) and read in a SpectraMax ID5 plate reader (Molecular Dynamics). Anisotropy values from the technical replicates were averaged and corrected by subtracting the value from a buffer control that contained no protein. Values from three independent experiments were plotted in GraphPad PRISM and fit to the Hill equation. For Pol-α/primase recruitment experiments, 6-FAM-labeled oligodT60 was pre-incubated with 2.5 nM of the respective RPA protein in the absence of supplemental NaCl. Then, Pol-α/primase was titrated into RPA-bound DNA mixture and incubated for 30 min before reading samples as described above. All samples were corrected for by subtracting a control well with no added Pol-α/primase.

### Unwinding assay

Unwinding assays were performed on 3.8 kb pUC19-ARS1 plasmids as described (12) with modifications. Briefly, 25 fmol of plasmid DNA was relaxed with 0.4 pmol Topo I for 30 min at 30°C. Then, Mcm2-7 loading was performed at 25°C by adding 45 nM ORC, 45 nM Cdc6 and 100 nM Mcm2–7/Cdt1 in 25 mM HEPES–KOH (pH 7.6), 10 mM magnesium acetate, 225 mM potassium glutamate, 2 mM DTT, 0.02% NP-40, 5% glycerol, 5 mM ATP, 20 mM phosphocreatine and 0.2 μg of creatine kinase for a total volume of 10 μl. After a 25 min incubation, 1.3 pmol of DDK was added and incubation was continued for a further 30 min. DNA unwinding was then initiated by adding 20 μl of firing-factor mix (0.6 pmol CDK, 1 pmol Sld3/7, 1 pmol Cdc45, 1.24 pmol Sld2, 0.8 pmol Dpb11, 5 pmol GINS, 0.06 pmol Mcm10, 0.6 pmol Pol ϵ, 0.4 pmol of Topo I and the indicated SSB amount) in 25 mM HEPES–KOH (pH 7.6), 10 mM magnesium acetate, 250 mM potassium glutamate, 1 mM DTT, 0.02% NP-40, 8% glycerol, 5 mM ATP, and 0.4 mg/ml BSA. After a 40 minute incubation at 25°C, the reaction was quenched with EDTA, Proteinase K and SDS as described (12). Samples were purified by phenol:chloroform extraction, ethanol precipitation, and run on a 1.5% agarose TAE gel at 1.5 V/cm for 16–20 h before staining with ethidium bromide and imaging.

### Replication assay

Replication assays were performed on a soluble 11.9 kb *ARS1*-containing DNA template (pMM068) that was linearized with NotI and purified by phenol:chloroform extraction and ethanol precipitation. Helicase loading was performed by incubating 10 μl reactions with 0.125 pmol DNA, 0.5 pmol ORC, 0.5 pmol Cdc6 and 1.25 pmol Mcm2-7/Cdt1 in a buffer containing 25 mM HEPES (pH 7.6), 10 mM MgOAc, 2 mM DTT, 100 mM KGlut, 20 mM phosphocreatine (PC), 5 mM ATP, 0.01% NP-40, 5% glycerol and 0.2 μg of creatine kinase (CK) for 25 min at 25°C with shaking on an Eppendorf Thermomixer at 1250 rpm. Then, 1.3 pmol DDK was added and the reaction was incubated for another 25 min at 25°C at 1250 rpm. After DDK phosphorylation, replication was initiated by adding 20 μl of a replication mix containing 1 pmol CDK, 1 pmol Sld3/7, 2.6 pmol Cdc45, 1.24 pmol Sld2, 0.8 pmol Dpb11, 5 pmol GINS, 0.02 pmol Mcm10, 0.6 pmol Pol ϵ, 2 pmol Pol α/primase, 0.6 pmol Ctf4, 0.5 pmol RFC, 0.4 pmol PCNA, 0.5 pmol Mrc1, 0.6 pmol Csm3/Tof1, 0.2 pmol Pol δ and the indicated SSB concentration in replication buffer (12.5 mM HEPES–KOH (pH 7.6), 5 mM magnesium acetate, 125 mM potassium glutamate, 1 mM DTT, 0.01% NP-40, 4% glycerol, 1.5 mM ATP, 10 mM phosphocreatine, 3 μg of creatine kinase, 0.2 mg/ml BSA, 100 μM rNTP, 10 μM dNTP and 10 μCi [α-P^32^]-dCTP). After a 60-minute incubation at 25°C while shaking at 1250 rpm, reactions were quenched with EDTA and unincorporated nucleotides were removed with Illustra MicroSpin G-50 columns (Cytiva). Samples were separated on a 0.6% alkaline agarose gel run at 20 V for 16 h in Alkaline Running Buffer (30 mM sodium hydroxide, 2 mM EDTA). Gels were dried onto Amersham Hybond-XL (GE Healthcare) and imaged and scanned using an Amersham Typhoon phosphorimager (Cytiva).

## RESULTS

### EcSSB, but not Gp32, substitutes for RPA during origin DNA unwinding

To study the role of RPA during eukaryotic DNA replication origin unwinding, we compared purified yeast RPA, *E. coli* SSB (EcSSB), and T4 bacteriophage Gp32 (Figure 1D). The ssDNA-binding properties of these proteins have been well characterized, however, it was important that we measured the relative ssDNA-binding affinities for our protein preparations. To estimate DNA binding affinities, we incubated varying concentrations of each protein with a fixed concentration of a fluorescently-labeled 30-nucleotide dT oligonucleotide (6FAM-dT_30_), then measured fluorescence polarization. To ensure equilibrium binding, all reactions were performed in high-salt conditions (54) and incubated for 30 min, after which we observed no further changes in anisotropy. Consistent with previously reported measurements (31,32,35,56), Gp32 showed the lowest binding affinity, followed by RPA, then EcSSB (Figure 1E and Supplemental Table S1). Importantly, all subsequent assays are performed under saturating concentrations for each SSB.

**Figure 2.**
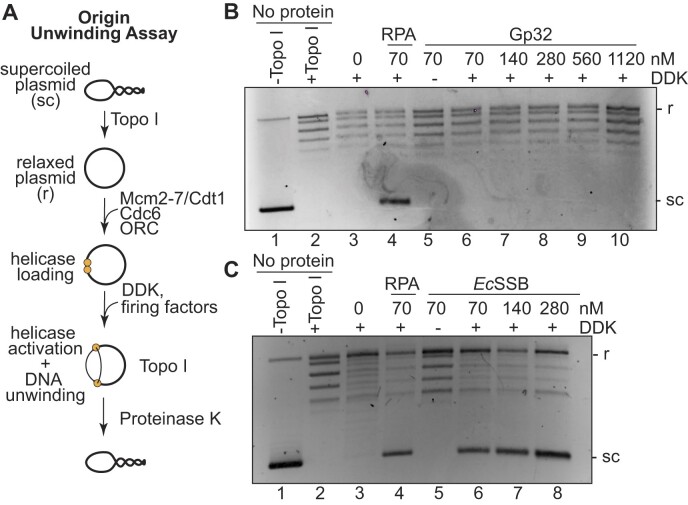
EcSSB, but not Gp32, can support Mcm2-7 unwinding of origin DNA. (**A**) Schematic of the origin DNA unwinding assay. (**B**) Origin DNA unwinding assays with a 2-fold titration series of Gp32 in the absence of RPA. (**C**) Origin DNA unwinding assay substituting the indicated concentrations of EcSSB for RPA. In both origin unwinding experiments, ‘no protein’ controls (lanes 1–2) contain only plasmid +/- Topoisomerase I (Topo I) to show migration of supercoiled (sc) and relaxed (r) plasmids.

Next, we tested the RPA requirements for origin activation using an *in vitro* origin DNA-unwinding assay using purified budding yeast proteins (12). We detected extensive origin-dependent DNA unwinding using a topologically-constrained circular *ARS1*-containing DNA template, onto which we loaded and activated Mcm2-7 in the presence of a topoisomerase (Figure 2A). The topoisomerase relieves supercoiling generated by DNA duplex unwinding. Reactions are terminated by rapid protein denaturation, removing all protein from the DNA. This treatment results in unwound DNA re-annealing in the absence of topoisomerase, generating negative supercoils that migrate faster in an agarose gel compared to the relaxed plasmid (Figure 2A). The topological changes observed are DDK-dependent, consistent with this kinase being required for assembly of the CMG helicase (Supplemental Figure S1 and Figure 2B, C, lane 5). As previously observed (12), the extensive origin DNA unwinding measured in this assay requires RPA (Figure 2B, C, lanes 3 versus 4). We note that this assay detects extensive origin unwinding, but not the initial, RPA-independent origin melting that results from the initial steps of helicase activation.

If extensive origin unwinding only requires the ssDNA-binding activity of RPA, then other SSBs should support eukaryotic DNA unwinding. When we tested Gp32 in the DNA-unwinding assay, we did not observe levels of DNA unwinding above that detected in the absence of any SSB (Figure 2B, lanes 6–10). Even increasing the concentration of Gp32 into the micromolar range did not restore DNA unwinding activity, eliminating the possibility that Gp32’s lower ssDNA-binding affinity explains the lack of DNA unwinding (Figure 2B). The inability of Gp32 to support origin unwinding suggests having a protein coat the exposed ssDNA is not sufficient for this process. Interestingly, substituting EcSSB in the unwinding assay supported robust DNA unwinding (Figure 2C, lanes 6–8). We observed similar levels of unwinding between RPA and EcSSB when provided in reactions at the same concentration (Figure 2C, compare lanes 4 and 6). Thus, EcSSB shares the properties of RPA that are required for eukaryotic replication origin unwinding.

### Multiple RPA ssDNA-binding domains are required to promote origin unwinding

The ability of EcSSB, but not Gp32, to substitute for RPA during origin unwinding has two interesting implications. First, simple ssDNA binding is not sufficient to promote extensive origin unwinding, suggesting other ssDNA-binding properties are important. Second, because EcSSB functions as well as RPA, specific interactions with the CMG helicase are not required for this event. Unlike the monomeric Gp32, RPA and EcSSB are both large, multimeric complexes containing a total of four DNA-binding domains, bind a larger ssDNA footprint than Gp32, and engage in dynamic associations with ssDNA (20,34). One or more of these shared features of RPA and EcSSB must be required for eukaryotic origin unwinding.

**Figure 3.**
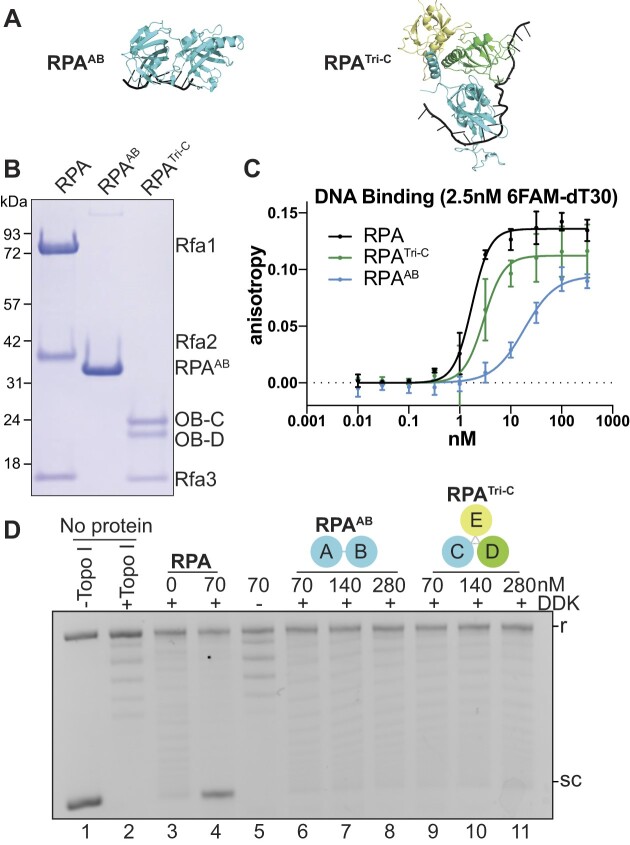
RPA subcomplexes containing two DNA-binding domains do not support origin DNA unwinding. (**A**) Structures of RPA^AB^ and RPA^TriC^subdomains (1JMC, 6I52, respectively). (**B**) Coomassie-stained SDS-PAGE gel of purified RPA, RPA^AB^ and RPA^Tri-C^. (**C**). Fluorescence polarization results of 2.5 nM 6-FAM oligo-dT30 incubated with indicated concentrations of RPA^AB^ (cyan; *K*_d,app_ ∼ 18.35 nM) or RPA^Tri-C^ (green; *K*_d,app_ ∼ 2.97 nM). The wild-type RPA results (*K*_d,app_ ∼ 1.7 nM) from Figure 1 are included for comparison. (**D**) Origin DNA unwinding assays with two-fold titration of RPA^AB^ or RPA^TriC^ (see also Figure 2 legend).

Since Gp32 contains only a single DNA-binding domain, we first tested whether the number of DNA-binding domains explained the different ability of Gp32 and RPA/EcSSB to facilitate origin unwinding. To this end, we generated RPA mutants containing two DNA-binding domains. RPA can be divided into two subdomains, each containing two DNA-binding domains that bind ssDNA with different properties. RPA^AB^ is composed of OB-A and OB-B domains, which are adjacent to one another in Rfa1 and together bind 8 nts of ssDNA in an approximately linear conformation (Figures 1A and 3A) (28). In contrast, the RPA trimerization core (RPA^Tri-C^) is a trimer made up OB-domains from three separate subunits (OB-C/D/E; only OB-C and OB-D bind ssDNA) that exerts a local bend in the 19 nt of bound ssDNA (Figures 1A and 3A) (27). These different DNA-binding properties contribute to different affinity for and dynamics on ssDNA.

We purified the RPA^AB^ and RPA^Tri-C^ subdomains and tested their ability to bind DNA and stimulate origin unwinding. When we tested these RPA mutants in fluorescence polarization assays, both RPA^AB^ and RPA^Tri-C^ displayed lower ssDNA-binding affinity compared to full-length RPA. RPA^AB^ showed ssDNA binding comparable to that of Gp32, whereas RPA^Tri-C^ DNA-binding affinity was between that of RPA and RPA^AB^ (Figure 3C and Supplemental Table S1). The relative affinities are consistent with recent studies on RPA ssDNA-binding dynamics (23,25). When we tested each two-ssDNA-binding-domain mutant in the origin unwinding assay, neither RPA^AB^ nor RPA^Tri-C^ stimulated DNA unwinding. As with Gp32, increasing the concentration of RPA^AB^ or RPA^Tri-C^ to compensate for their lower ssDNA affinity did not restore origin DNA unwinding (Figure 3D and Supplemental Figure S2). We conclude that an ssDNA-binding protein with two DNA-binding domains is not sufficient to promote origin DNA unwinding.

**Figure 4.**
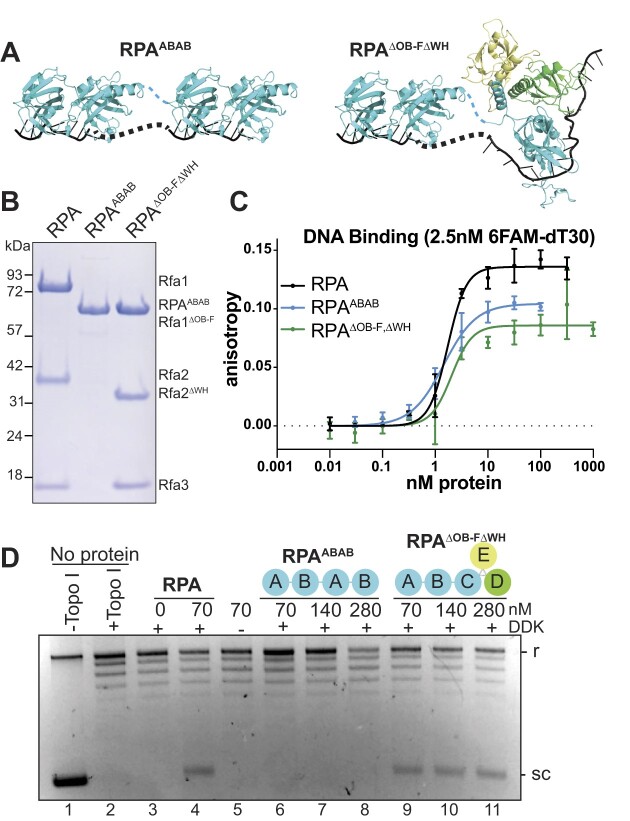
A high-affinity four DNA-binding domain RPA mutant is not sufficient for origin unwinding. (**A**) Diagrams of RPA^ABAB^ and RPA^ΔOBΔWH^ mutants. (**B**) Coomassie-stained SDS-PAGE gel of purified wild-type and mutant RPAs. (**C**) Fluorescence anisotropy results of 2.5 nM 6FAM-oligo-dT30 incubated with indicated concentrations of RPA^ABAB^ (*K*_d,app_ ∼ 1.45 nM) or RPA^ΔOB-FΔWH^ (*K*_d,app_ ∼ 2.04 nM). The wild-type RPA results (*K*_d,app_ ∼ 1.7 nM) from Figure 1 are included for comparison. (**D**) Origin DNA unwinding assay with two-fold titration of RPA^ABAB^ or RPA^ΔOB-FΔWH^.

Because both RPA and EcSSB include four DNA-binding domains, we asked if the higher avidity or larger binding site of this number of ssDNA-binding domains is required to promote origin unwinding. To test this possibility, we made an artificial RPA mutant containing two copies each of the OB-A and OB-B domains in a single polypeptide (RPA^ABAB^). The two copies of OB-A and OB-B are connected by the native linker that separates OB-B from OB-C (Figure 4A, B). Structural evidence shows the OB-A and OB-B domains bind 8 nt of ssDNA (28), suggesting RPA^ABAB^ can bind at least 16 nt and perhaps more due to the flexible linker between the two AB domains. This RPA mutant showed ssDNA binding that is comparable to wild-type RPA (Figure 4C and Supplemental Table S1). When tested in DNA-unwinding assays, however, RPA^ABAB^ failed to promote extensive origin DNA unwinding (Figure 4D, lanes 6–8). These data support the conclusion that neither ssDNA-binding affinity nor number of DNA-binding domains explain the SSB requirement during origin unwinding.

To test whether origin unwinding requires the endogenous, multimeric context of RPA, we generated an RPA mutant, RPA^ΔOB-FΔWH^, that contains all four DNA-binding domains but lacks the known RPA protein-interaction domains, Rfa1-OB-F and Rfa2-WH (Figure 4A-B). These domains interact with DNA repair and replication proteins, such as Pol-α/primase and Dna2, but are not involved in DNA binding (21,47,57–63). Indeed, we found this RPA mutant bound DNA with near wild-type affinity (Figure 4C and Supplemental Table S1). In contrast with the RPA^ABAB^ mutant, RPA^ΔOB-FΔWH^ supported robust DNA unwinding at all concentrations tested (Figure 4D, lanes 4 versus 9–11). Together, these RPA mutant results indicate that extensive DNA unwinding at replication origins requires the specific arrangement of DNA-binding domains in RPA. Combined with our finding that EcSSB functions during origin unwinding, these studies support a model in which DNA bending and/or dynamic changes in ssDNA-binding modes are required for origin DNA unwinding.

### Replication elongation requires the RPA OB-F and WH domains

**Figure 5.**
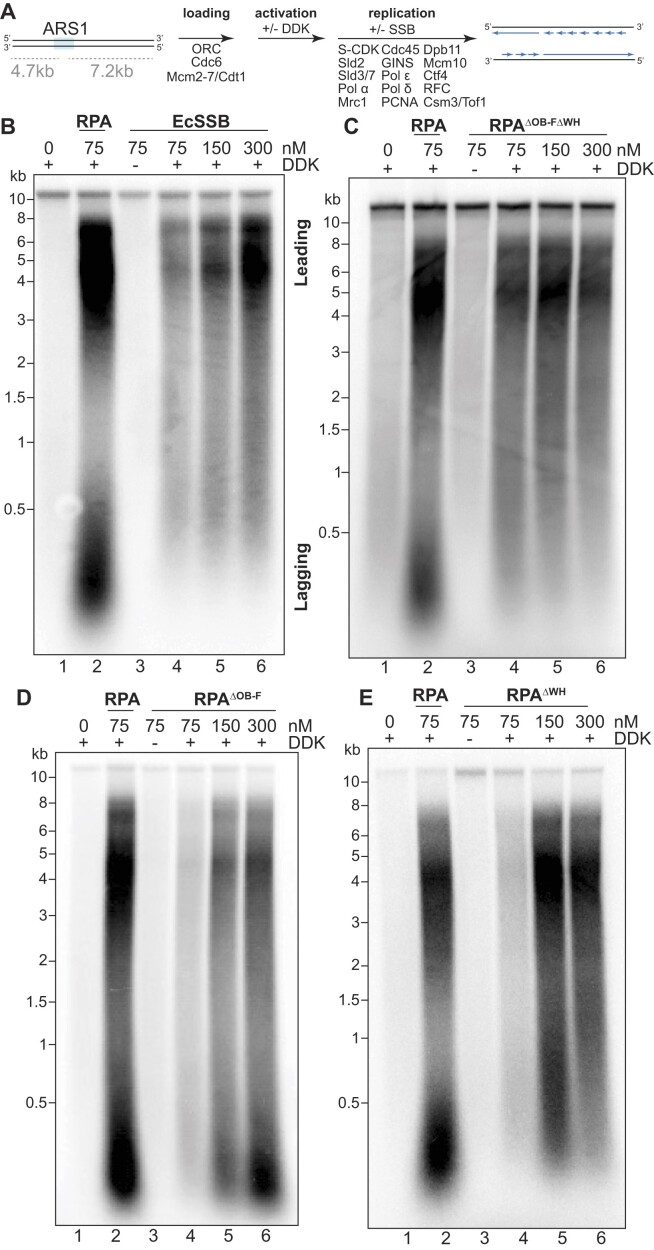
RPA OB-F and WH domains control replication elongation. (**A**) Schematic of the reconstituted DNA replication assay. (B–E) *In vitro* replication with a two-fold titration series of (**B**) EcSSB, (**C**) RPA^ΔOB-FΔWH^, (**D**) RPA^ΔOB-F^ or (**E**) RPA^ΔWH^ substituting for RPA. Locations of normal leading- and lagging-strand product distributions are labeled in (B). As with the origin DNA unwinding assay, replication is dependent on RPA (compare lanes 1 and 2) and DDK (compare lanes 3 and 4).

We next asked whether ssDNA-binding proteins that support origin unwinding also support DNA synthesis. To monitor replication initiation and elongation, we utilized an *in vitro* reconstituted DNA replication assay on linearized *ARS1*-containing plasmid DNA (Figure 5A) (9,14). Nascent DNA strands are monitored by incorporation of radiolabeled dNTPs followed by separation by alkaline gel electrophoresis. Omission of the flap endonuclease (Fen1) and DNA ligase from the reactions allows distinction of long leading-strand synthesis products (3000–6000 nts) from shorter lagging-strand products (100–500 nts) (Figure 5) (9).

We found that DNA synthesis has distinct RPA requirements compared to DNA unwinding. As previously reported (9), we saw no DNA replication without RPA, and addition of RPA led to robust, DDK-dependent leading- and lagging-strand synthesis (Figure 5B–D, compare lanes 1–4). Consistent with its defect in promoting origin unwinding, addition of Gp32 did not support any DNA synthesis (Supplemental Figure S3A). When EcSSB was substituted for RPA, however, we observed a distinct pattern of replication products. When supplied at the equivalent concentration of RPA (75 nM), EcSSB supported weak DNA-synthesis activity (Figure 5B, lane 4). Thus, although this EcSSB concentration supports robust DNA unwinding (Figure 2), it showed a strong defect in initiating and/or elongating nascent DNA. Increasing the EcSSB concentration resulted in a corresponding increase in DNA synthesis, however, the distribution of leading- and lagging-strand products was strikingly different than that observed with RPA. With EcSSB, we consistently observed a much larger fraction of long products (Figure 5B, lanes 5–6), suggesting that lagging-strand DNA synthesis is defective. This could be due to fewer lagging-strand synthesis products or to longer Okazaki fragments that comigrate with the leading-strand products. In either scenario, RPA performs one or more essential functions at the eukaryotic replication fork that cannot be complemented by EcSSB.

The failure of EcSSB to support normal leading- and lagging-strand replication suggests that replication requires direct contacts between RPA and the replisome. Interestingly, when we tested the RPA^ΔOB-FΔWH^ mutant that lacks both of RPA’s protein-interaction domains but binds ssDNA with wild-type affinity (Figure 4), we observed results that were very similar to experiments with EcSSB (Figure 5C). These results suggest that the OB-F and WH domains control important aspects of DNA replication.

To investigate this connection further, we generated deletion mutants lacking either the OB-F or the WH domain. Like RPA^ΔOB-FΔWH^, these mutants both support robust origin unwinding at all concentrations tested (Supplemental Figure S3B, C). Strikingly, both RPA^ΔOB-F^ and RPA^ΔWH^ have strong replication defects when supplied at the same concentration as RPA (Figure 5D, E, lanes 2 versus 4), showing that they each are critical for efficient replication initiation or elongation. Increasing the concentration of either mutant protein increased nucleotide incorporation but resulted in abnormal patterns of leading- and lagging-strand replication products. Higher concentrations of RPA^ΔOB-F^ led to an accumulation of short products comigrating with lagging-strand products (Figure 5D, lanes 4–6). In contrast, increasing amounts of RPA^ΔWH^ produced a preponderance of longer replication products that lacked the shorter Okazaki fragment lengths. This pattern was similar to that in reactions with EcSSB or with RPA^ΔOB-FΔWH^ (Figure 5E, lanes 4–6). These results show that the two RPA protein-interaction domains control different properties of DNA replication initiation or elongation.

Defects in priming would more strikingly affect lagging-strand synthesis because of the need to constantly re-initiate DNA synthesis on this strand. Because RPA affects multiple Pol-α/primase activities, we considered that altering RPA–Pol-α/primase interactions may explain the defects in replication product size distribution. To test this, we first increased the concentration of Pol-α/primase in the reconstituted replication assay. Because reactions with the RPA mutants had low amounts of DNA synthesis in our standard assay conditions (Figure 5D-E, lane 2 versus 4–6), we used a higher concentration of RPA (150nM) in these experiments.

**Figure 6.**
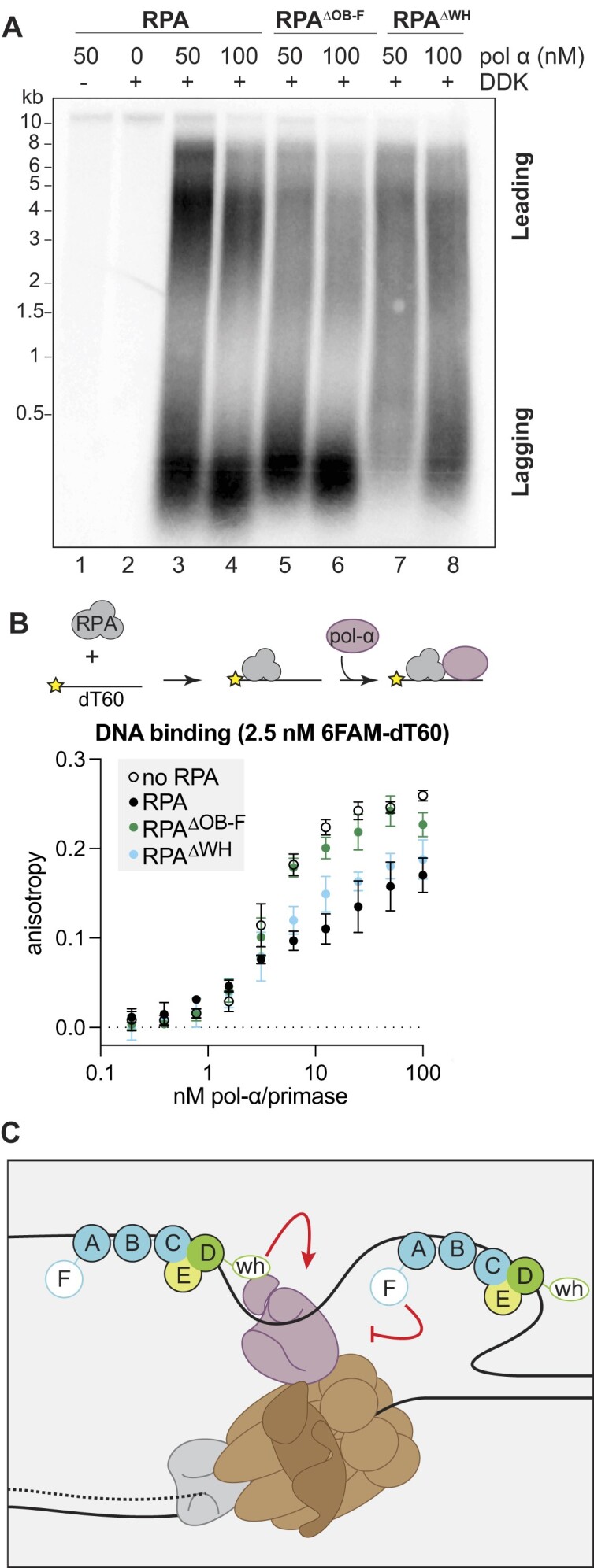
Pol-α/primase mediates the defects of the OB-F and WH deletions. (**A**) Titration of Pol-α/primase in *in vitro* replication assays containing 150 nM of the respective RPA protein. (**B**) Fluorescence anisotropy of Pol-α/primase titration with a 2.5 nM 6FAM-oligo-dT60 pre-incubated with an equimolar amount of the indicated RPA. (**C**) Diagram of Pol-α/primase (purple) with CMG (brown) and RPA at a replication fork with OB-F and WH interactions.

Increasing Pol-α/primase concentration resulted in distinct changes in replication products for the different RPAs. In reactions with wild-type RPA, additional Pol-α/primase resulted in a higher prevalence of short products relative to the long leading-strand products. Additionally, the lagging-strand products were slightly shorter (Figure 6A, lanes 3–4, and Supplemental Figure **S**4). This trend resembles the defect we have observed with the RPA^ΔOB-F^ mutant, with an increase in short products at the expense of the longer products. Interestingly, in reactions with RPA^ΔOB-F^, increasing Pol-α/primase also shortened the length of the lagging-strand products but did not strikingly alter the leading- and lagging-strand distribution (Figure 6A, lanes 5–6 and Supplemental Figure S4). This finding is consistent with negative role for the OB-F domain in Pol-α/primase function. In contrast, reactions with RPA^ΔWH^ showed partial restoration of short lagging-strand products without any major changes in the longer leading-strand products (Figure 6A, lanes 7–8), a result that is consistent with a positive regulatory role of this domain. Together these results are consistent with the RPA OB-F and WH domains negatively and positively regulating the priming of DNA synthesis, respectively.

Next, we asked whether the RPA mutants affect Pol-α/primase binding to ssDNA. To this end, we measured Pol-α/primase binding on naked ssDNA or ssDNA pre-incubated with an equimolar amount of RPA using fluorescence polarization (Figure 6B). By using a longer oligonucleotide (6FAM-dT60), we can observe binding of RPA and Pol-α/primase on the same ssDNA molecule. When we titrated Pol-α/primase, we observed a concentration-dependent increase in anisotropy. The maximum anisotropy was notably higher on naked DNA than on RPA/ssDNA complexes (Figure 6B), consistent with RPA reducing Pol-α/primase association with the oligo-dT60 template. When we pre-incubated ssDNA with RPA^ΔWH^, there was no significant difference from pre-incubation with WT RPA. However, when ssDNA was pre-incubated with RPA^ΔOB-F^, Pol-α/primase binding matched that of the naked DNA (Figure 6B). These results show that the removal of the OB-F domain allows more Pol-α/primase to bind to an ssDNA template coated with RPA, supporting a negative regulatory role of this domain. This observation is consistent with our observation of increased lagging-strand synthesis in replication reactions with RPA^ΔOB-F^ (Figures 5D and 6A). Overall, our RPA mutants have revealed two distinct activities that are crucial for appropriate replication fork assembly or function.

## DISCUSSION

By substituting other SSBs or mutant versions of RPA into different replication assays, we have gained important insights into the function of RPA during eukaryotic replication initiation and elongation. We found that RPA requires two protein-interaction domains, OB-F and WH, to coordinate DNA synthesis at the eukaryotic replication fork. Interestingly, these domains are dispensable for origin unwinding. Instead, we find that a particular arrangement of ssDNA binding domains found in RPA and EcSSB, but not Gp32, is required for this event.

### RPA function during helicase activation and unwinding

Previous work has shown evidence of CMG activation and translocation on ssDNA in the absence of RPA (12). These data suggest that the purpose of RPA is to simply keep the strands separated after CMG passes. However, our results indicate that preventing strand re-annealing is not the sole purpose of RPA during origin unwinding (Figures 2–4). We find that, even at very high concentrations, Gp32 does not support extensive DNA unwinding in our assays. Additionally, Gp32 fails to support any DNA synthesis (Supplemental Figure S3), suggesting that the DNA is insufficiently unwound to support replisome assembly. This is despite the fact that Gp32 participates in rapid cooperative ssDNA binding (64) with a slow off-rate. Importantly, single-molecule evidence indicates that Gp32 binding prevents re-annealing of ssDNA (65), and Gp32 is known to stabilize ssDNA regions to allow primer binding in plasmid DNA (66). In addition, the RPA^ABAB^ mutant has the same ssDNA binding affinity as RPA, yet fails to promote extensive origin DNA unwinding, further supporting our conclusion that RPA contributes to more than preventing strand re-annealing during the transition to extensive origin DNA unwinding. Based on these findings, we argue that RPA must improve the efficiency of CMG activation or translocation on ssDNA.

There are several features of the three proteins that stimulated extensive origin unwinding (RPA, EcSSB and RPA^ΔOB-FΔWH^) that are not shared by the small monomeric Gp32 or the RPA mutants tested. First, multiple dynamic ssDNA-binding modes contribute to RPA (23,25) and EcSSB (36) function. These multiple ssDNA-binding modes are important for binding various DNA structures, such as single-strand gaps, bubbles and ss/dsDNA junctions (67). Second, not only are RPA and EcSSB larger than Gp32 by molecular weight, their structures also contain clustered OB-fold domains (Figure 1A–C). Finally, structural evidence shows that both RPA and EcSSB can induce ssDNA-bending (Figure 1A and C, (27,29,33)). The single binding domain of Gp32 cannot reproduce the ssDNA-binding dynamics, clustered OB-folds, or ssDNA bending exhibited by EcSSB and RPA.

A combination of these three properties could contribute to two important requirements for origin activation. First, RPA ssDNA-binding dynamics may improve the efficiency of CMG activation by supporting the DNA remodeling steps required as the helicase transitions from encircling dsDNA to encircling ssDNA. For example, RPA binding to partially extruded DNA could facilitate strand extrusion (Supplemental Figure S5B). Secondly, because the Mcm2-7 helicases are loaded in a head-to-head conformation (2,4), they must pass each other on opposite strands of DNA before unwinding DNA bidirectionally from the replication origin. RPA could promote this process by eliminating steric barriers, through its physical size, effects on ssDNA conformation, or both (Supplemental Figure S5C). Although CMG translocation on ssDNA is detectable in the absence of RPA (12), the efficiency of this transition is low. We propose that RPA increases the efficiency of this transition as is observed in DNA unwinding assays (Figure 2 and (12)).

Finally, RPA binding to the ssDNA strand excluded from CMG could stimulate helicase processivity in a way that promotes extensive origin unwinding. Although RPA modulates the activity of other helicases through direct interactions (62,68), this has not been observed for the CMG helicase. Indeed, the ability of EcSSB and RPA^ΔOB-FΔWH^ to substitute for RPA indicates that direct protein-protein interactions between RPA and the helicase are not required. Instead, we consider that RPA stimulates origin unwinding by interacting with the excluded strand during CMG translocation (Supplemental Figure S5D). Structural studies of the CMG complex found that the excluded strand interacts with the helicase central pore (69–71). Recent evidence suggests this interaction leads to CMG stalling, and RPA binding to the excluded strand stimulates the rate of the CMG helicase (19). Similarly, studies of the *E. coli* replicative helicase, DnaB, that showed applying tension to the excluded strand, but not the encircled strand, stimulates helicase activity (72). In contrast, a similar study using the T4 bacteriophage replicative helicase, Gp41, demonstrated that tension on the excluded strand inhibited DNA unwinding (73). If this type of tension on the excluded strand is important for CMG stimulation, these data would explain the ability of EcSSB, but not T4 Gp32 to function in the unwinding assay.

### Replication requires the RPA OB-F and WH domains

Specific RPA interactions are required at the eukaryotic replication fork. When substituted for RPA, both EcSSB and RPA^ΔOB-FΔWH^ displayed dramatically decreased DNA synthesis in reconstituted replication assays, suggesting defects in nascent strand initiation or elongation (Figure 5B, C and Supplemental Figure S3D). At higher EcSSB or RPA^ΔOB-FΔWH^ concentrations, DNA synthesis increased, but the products displayed altered distributions of leading- and/or lagging-strand products. Thus, in addition to overall DNA synthesis defects, our results show that specific interactions between RPA and the eukaryotic replication machinery are required for appropriate replication fork function. This is consistent with previous observations in SV40 replication, where neither EcSSB nor yeast RPA can efficiently substitute for human RPA (42,44). Similarly, the bacterial replisome requires specific interactions with the EcSSB C-terminal tails (74). New structural data of the eukaryotic replisome shows Pol-α/primase directly contacts the CMG helicase (75). These replisome structures were assembled without RPA, raising the possibility that RPA facilitates Pol-α/primase incorporation into the replisome. Such a function could explain the reduced DNA synthesis in replication reactions with EcSSB or the RPA protein-interaction domain mutants.

A previous study observed that EcSSB supports only Pol ϵ-dependent rolling-circle replication products in reconstituted yeast replication assays on circular DNA templates (76). Thus, Pol ϵ synthesis may explain the longer replication products and absence of short characteristic Okazaki fragments in reactions with EcSSB or RPA^ΔOB-F,ΔWH^ on our linear templates. These findings suggest that the OB-F and WH domains likely regulate Pol-α/primase and/or Pol δ activity at the replication fork. An alternative explanation is that the long products observed consist of long Okazaki fragments, possibly due to decreased priming on the lagging strand.

Consistent with the Rfa2 WH domain requirement in nascent strand initiation or Pol-α/primase recruitment to the replisome, increasing Pol-α/primase concentrations partially restored the synthesis of short replication products (Figure 6A). This result suggests that the Rfa2 WH domain facilitates Pol-α/primase binding or activity on ssDNA. Consistent with this hypothesis, previous studies found that deletion of the Rfa2-WH domain reduced RPA interaction with Pol-α/primase (77). We can also gain insight from structures of the conserved RPA-like CST complex with Pol-α/primase in its active conformation. These structures reveal direct contacts between Pol-α/primase and the Stn1 winged-helix domains, which are analogous to but divergent from the Rfa2-WH domain, and these contacts are proposed to promote Pol-α/primase activity and processivity (78,79).

Our RPA^ΔOB-F^ results implicate an important role for the Rfa1 OB-F domain in regulation of lagging-strand synthesis. We observed that replication assays with this mutant had weak leading-strand synthesis and accumulated Okazaki fragments that were shorter than those in control reactions (Figure 5D and Supplemental Figure S3D). Interestingly, increasing Pol-α/primase concentrations in reactions with WT RPA displayed a similarly skewed leading:lagging product distribution with shorter Okazaki fragments (Figure 6A). This suggests that excess Pol-α/primase activity could explain the results we have observed. In support of this hypothesis, we observed that RPA^ΔOB-F^ lacked the ability to limit Pol-α/primase ssDNA binding that we observed with full-length RPA (Figure 6B). Such a negative regulatory function for RPA could play a critical role in determining the frequency of lagging-strand priming given the recent finding suggesting that Pol-α/primase associates continuously with the replisome (75).

It is also possible that this negative regulation of Pol-α/primase is necessary for polymerase switching. Failure of this activity could prevent or delay handoff from Pol-α/primase to the more processive Pol δ and/or Pol ϵ, affecting both leading and lagging synthesis. Indeed, replication reactions with RPA^ΔOB-F^ do not recover leading-strand synthesis, even in the presence of excess Pol-α/primase (Figure 6A). Similarly, SV40 replication assays performed without the Pol δ processivity clamp PCNA display only short replication products (59), suggesting that accumulation of short products may be due to failure of polymerase switching. Indeed, genetic data supports a role of the OB-F domain in regulating not only Pol-α/primase but also Pol δ. Mutations in the Rfa1 OB-F domain are synthetic lethal with mutant alleles of Pol-α/primase, Pol δ and RFC (80,81).

Overall, our results reveal that the Rfa1 OB-F and Rfa2 WH domains each regulate a distinct step of nascent strand initiation or elongation. A function of the WH domain in stimulating priming activity and OB-F domain in negatively regulating this event fits with our analysis of the double and single mutants. We found that the WH deletion mimics the double mutant, whereas the OB-F mutant shows a distinct phenotype, consistent with the WH domain stimulating an activity that is required before the activity regulated by the OB-F domain. Future studies involving assays for specific steps of replication initiation will be required to determine the precise mechanism by which the OB-F and WH domains regulate replication fork function.

## Supplementary Material

gkad765_Supplemental_File

## Data Availability

The data underlying this article will be shared on reasonable request to the corresponding author.

## Notes

Present address: Alexandra M. Pike, Department of Biology, Oberlin College, Oberlin, OH 44074, USA.
