# Supplementary material for: Distinct RPA functions promote eukaryotic DNA replication initiation and elongation
Source: Nucleic Acids Res. 2023 Sep 22;51(19):10506–18. doi: 10.1093/nar/gkad765 (PMC10602884; doi:10.1093/nar/gkad765)
Supplement: gkad765_Supplemental_File [file gkad765_supplemental_file.docx]

# Supplemental Information


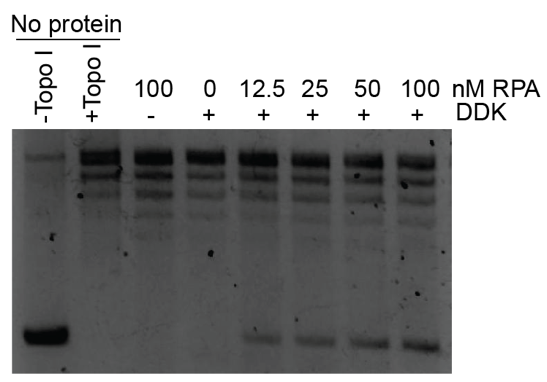


**Supplemental Figure 1**. RPA titration in the plasmid unwinding assay shows that unwinding is DDK-specific unwinding and requires sufficient concentrations of RPA.


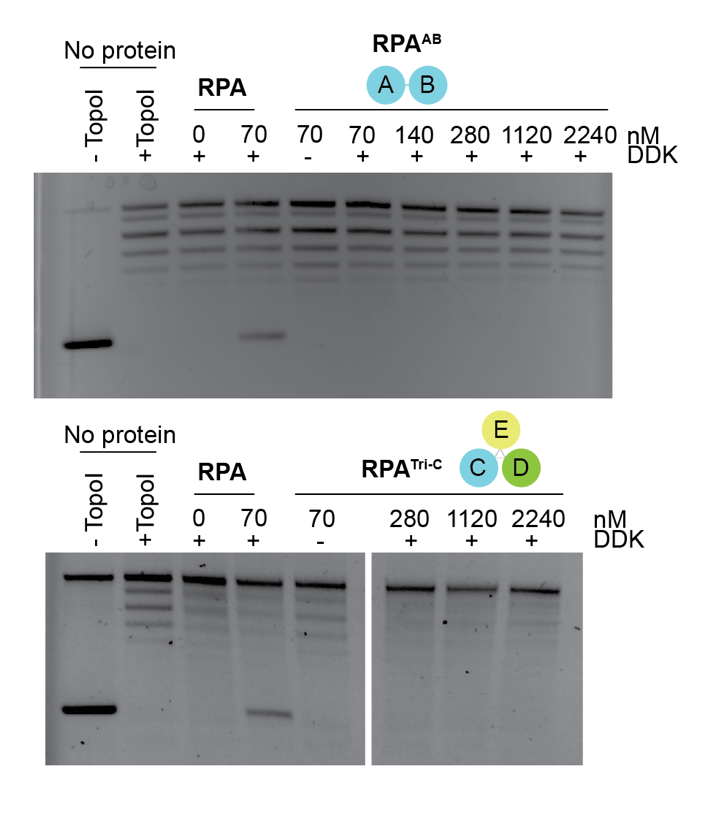


**Supplemental Figure 2**. Extended titration series of RPA AB (top) and Tri-C (bottom) mutants going up to 2.24 µM showed no observable DNA unwinding.


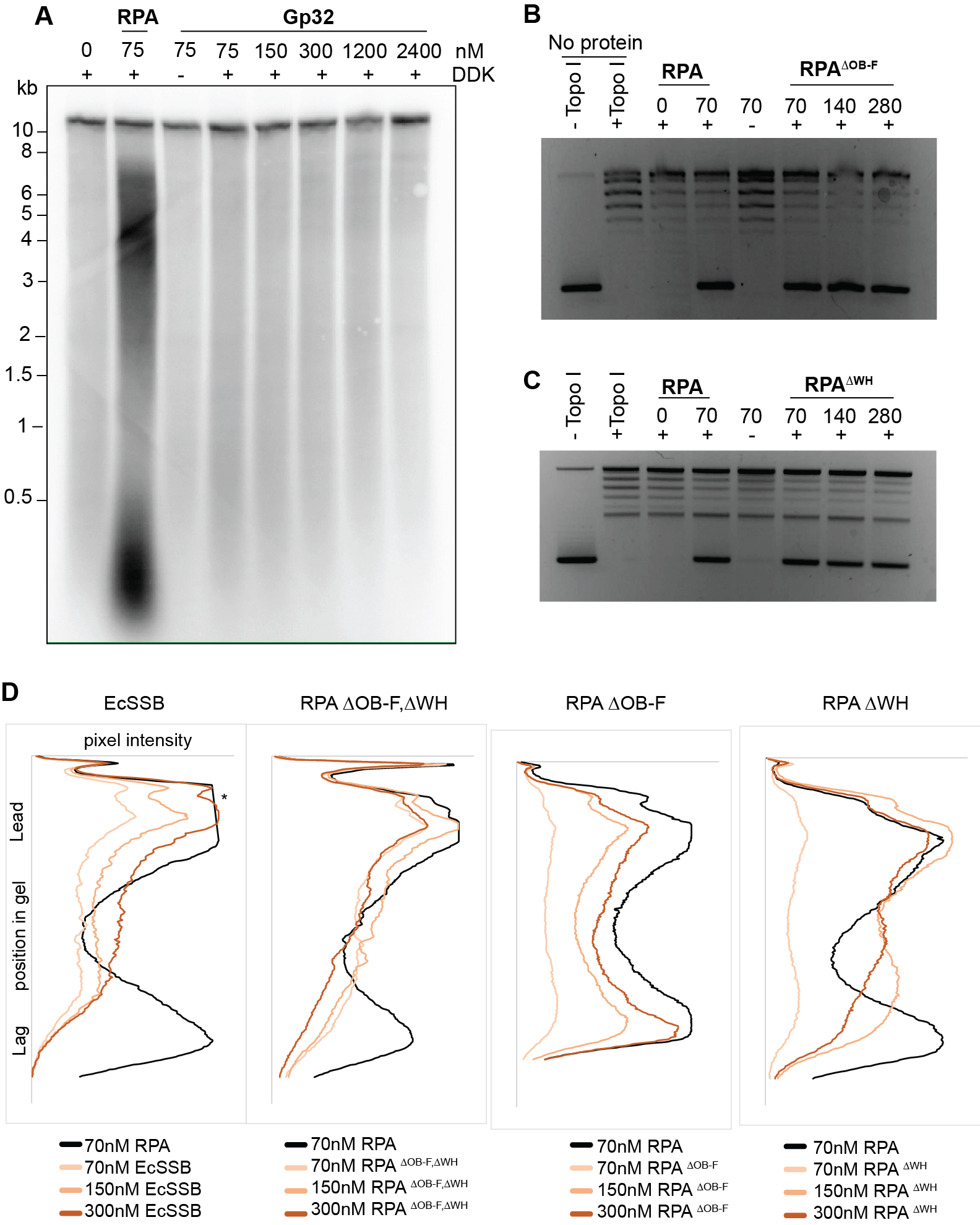


**Supplemental Figure 3**. A) Gp32 does not support DNA replication in eukaryotic replication assays. B-C) Unwinding assays with RPA single domain deletions show that, like the double mutant, the single domain deletions support DNA unwinding. D) Densitometry analysis of the replication assays in Figure 5(*, saturated pixels).

**Supplemental Figure 4**. Densitometry analysis of the reconstituted replication assay shown in Figure 6A. Pixel intensity is plotted against the position in the gel. Light blue, 50nM Pol-α/primase; dark blue, 100 nM Pol-α/primase. Asterisks in the WT RPA plot highlight the shift in the lagging strand peak.

**
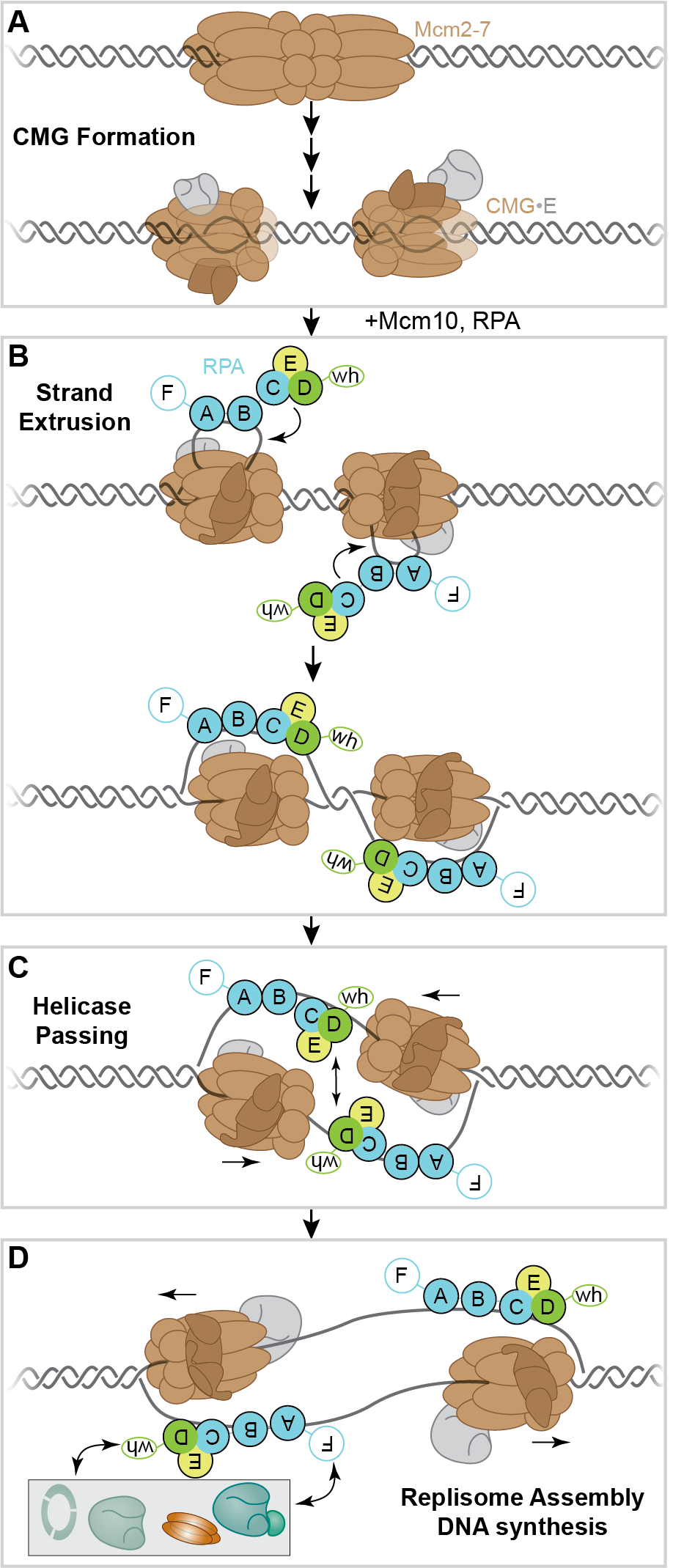
**

**Supplemental Figure 5.** Cartoon of the stages of CMG activation and replisome assembly. See discussion for details.

**Supplemental Table 1**. Apparent Kd values and 95% confidence intervals from anisotropy data fit to the Hill Equation. Note that these are not meant to be definitive measurements, particularly as the binding is complex due to multiple binding site cooperativity (as reflected in the Hill coefficient). These values are reported for relative comparison between the proteins assayed in this study.

| Protein | Kd,app | 95% CI | Hill Coefficient | Figure |
| --- | --- | --- | --- | --- |
| RPA | 1.729 | 1.480 to 2.015 | 2.460 | 1,3,4 |
| EcSSB | 0.114 | 0.08112 to 0.1484 | 3.430 | 1 |
| Gp32 | 18.670 | 15.25 to 23.03 | 1.911 | 1 |
| RPA-AB | 18.350 | 14.00 to 24.69 | 1.419 | 3 |
| RPA-TriC | 2.971 | 2.133 to 4.648 | 2.215 | 3 |
| RPA-ABAB | 1.450 | 1.182 to 1.794 | 1.405 | 4 |
| RPA-∆OB-F∆WH | 2.047 | 1.402 to 3.496 | 2.233 | 4 |

**Supplemental Table 2**. RPA Expression Plasmids. Plasmid name is given along with information about the coding region for each respective RPA subunit.

| Plasmid Name | RPA Coding Regions | | | Source |
| --- | --- | --- | --- | --- |
|  | Rfa1 | Rfa2 | Rfa3 |  |
| pRS303-Gal1,10-CBP-Rfa1/Gal4 | CBP-Rfa1 | -- | -- | yAE31 |
| pRS306-Gal1,10-Rfa2/Rfa3 | -- | Rfa2 | Rfa3 | yAE31 |
| pRS303-Gal1,10-CBP-Rfa1∆OB-F/Gal4 | CBP-Rfa1(131-621) | -- | -- | this study |
| pRS306-Gal1,10-3xFlag-Rfa2∆WH/Rfa3 | -- | 3xFlag-Rfa2(1-202) | Rfa3 | this study |
| pET3aTr_OB-AandB | CBP-Rfa1(186-289) | -- | -- | this study |
| p11d-scTriC (OB-C/D/E) | Rfa1(442-621) | Rfa2(232-182)-MxeGyrA intein-CBD-6xHis | Rfa3 | this study |
| pET3aTr_OB-ABAB | CBP-Rfa1(186-441)-GGGGGG-Rfa1(186-289) | -- | -- | this study |

**Supplemental Table 3**. Yeast Strains

| Strain | Additional Information | Genotype | Source |
| --- | --- | --- | --- |
| yRH101 | Base Strain | MATa ade2-1 trp1-1 leu2-3,112 his3- 11,15 ura3-1 can1-100  bar1::HisG lys2::HisG pep4Δ::KanMX | Bell Lab |
| yAE31 | RPA expression strain (CBP-Rfa1) | MATa ade2-1 ura3-1 his3-11,15 trp1-1 leu2-3,112 can1-100 bar1::hyg pep4::kanMX HIS3 GAL1,10 CBP-TEV-RFA1opt + GAL4 URA3 GAL1,10 RFA2opt + RFA3opt | Diffley Lab (Yeeles 2015) |
| yAP18 | RPA ∆OB-F, ∆WH expression strain | MATa ade2-1 ura3-1 his3-11,15 trp1-1 leu2-3,112 can1-100 bar1::HisG lys2::HisG pep4Δ::KanMX HIS3 GAL1,10 CBP-TEV-RFA1∆OB-F + GAL4 URA3 GAL1,10 3xFlag-RFA2 + RFA3 | This Study |
| yAP05 | RPA ∆OB-F  expression strain | MATa ade2-1 ura3-1 his3-11,15 trp1-1 leu2-3,112 can1-100 bar1::HisG lys2::HisG pep4Δ::KanMX HIS3 GAL1,10 CBP-TEV-RFA1∆OB-F + GAL4 URA3 GAL1,10 RFA2 + RFA3 | This Study |
| yAP17 | RPA ∆WH  expression strain | MATa ade2-1 ura3-1 his3-11,15 trp1-1 leu2-3,112 can1-100 bar1::HisG lys2::HisG pep4Δ::KanMX HIS3 GAL1,10 CBP-TEV-RFA1 + GAL4 URA3 GAL1,10 3xFlag-RFA2-∆WH + RFA3 | This Study |
